# Proximal femoral fracture in a man resulting from modern clipless pedals: a case report

**DOI:** 10.1186/1752-1947-5-219

**Published:** 2011-06-07

**Authors:** James Parker, Neil Patel, Ganesh Devarajan

**Affiliations:** 1 Hull and East Yorkshire NHS Trust, Hull Royal Infirmary, Anlaby Road, Hull, HU3 2JZ, UK

## Abstract

**Introduction:**

The use of clipless pedals amongst recreational cyclists has become increasingly popular in recent years. We describe a hip fracture, that was sustained due to inadequate set up of such pedals. To the best of our knowledge, this has only been described once before, and this was in the non-English language medical literature.

**Case Report:**

A 38-year-old Caucasian man who was a club cyclist sustained a displaced intracapsular fracture of the hip whilst cycling. As a direct result of the incorrect set-up of his clipless pedals he was unable to release his feet whilst slowing to a halt. This resulted in a loss of balance and subsequent fall with a direct impact onto his left hip. The resulting fracture was managed successfully with early closed reduction and fixation. At six month review he was walking unaided without pain but, as yet, has been unable to return to cycling.

**Conclusion:**

This case highlights the dangers of clipless pedals even in experienced cyclists, and underlines the importance of proper information for their correct setup to minimise the risk of potentially serious injuries, especially in the region of the hip.

## Introduction

Intra-capsular fractures of the femoral neck are extremely common in the elderly population and may be associated with relatively minor trauma. In the younger population, however, intra-capsular fractures are usually the result of high energy trauma with serious consequences of avascular necrosis of the femoral head.

We report a case of a displaced intra-capsular hip fracture in an otherwise fit and well 38-year-old Caucasian man as a direct result of his bicycle pedals being set too tight.

## Case presentation

**Figure 1:**
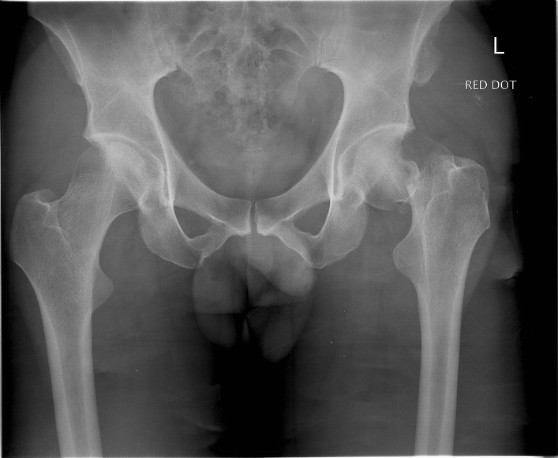
**Pelvic radiograph view showing fracture to his left proximal femur**.

**Figure 2:**
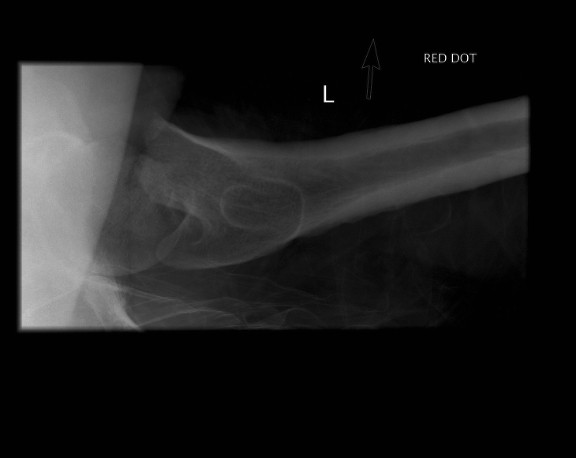
**Radiograph showing lateral view of his fractured left proximal femur**.

A previously healthy 38-year-old Caucasian man and competitive amateur cyclist sustained a displaced fracture of his left femoral neck following a fall from his racing bicycle whilst at rest. Having slowed gradually to a halt, our patient attempted to unclip his feet from the pedals. He was unable to unclip his feet and when the bicycle slipped on some ice, he was unable to remove his feet to steady himself. As a result he sustained a direct trauma to his left hip, resulting in a displaced intra-capsular fracture of the right femoral neck (Figure 1, 2).

**Figure 3:**
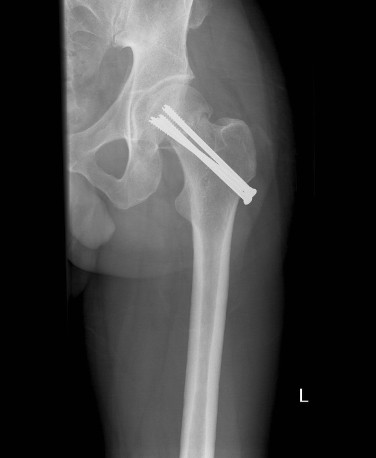
**Radiograph of his left hip six months after surgery**.

**Figure 4:**
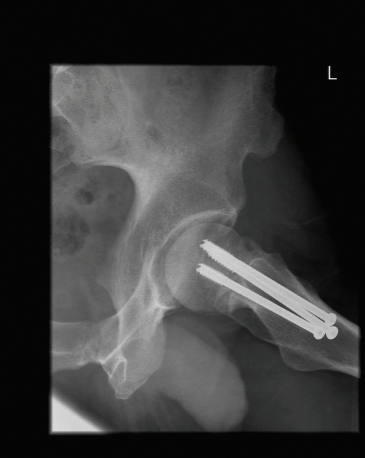
**Radiograph of his left hip six months after surgery**.

Once other injuries had been excluded and adequate imaging had been obtained, our patient was admitted to our orthopedic ward and a closed reduction and internal fixation was performed later the same day. Reduction was obtained using the Leadbetter maneuver [1], and fixation performed with three 6.5 mm cannulated screws (Figure 3, 4). The surgical treatment was completed within 12 hours of the injury. Subsequent to the fixation our patient progressed well with no immediate complications and was discharged two days following the injury. Toe touch weight bearing was commenced for a period of six weeks and, following satisfactory radiographs, partial weight bearing was allowed for a further six weeks.

Although our patient had no risk factors for osteoporosis, given the relatively low energy of the injury, a bone density scan was performed along with other metabolic bone disease screening tests. These were all within normal limits.

At a subsequent review four months after the injury, our patient had no pain in the hip, a good range of movement and is walking unaided. Radiographic appearances are satisfactory and show no evidence of avascular necrosis. Regular clinical and radiographic review is planned until two years after the injury.

## Discussion

To ensure efficient transfer of power from the rider to the cycle during cycling, good binding of the feet to the pedal is beneficial to prevent the foot from slipping and to allow a smooth cadence. Traditionally, this involved the use of cycling shoes with rudimentary cleats strapped into a pedal with toeclips. This, however, often required the cyclist to strap their feet so tightly that they had to physically loosen the straps by hand to disengage the pedal making it almost useless for anyone but the most proficient cyclists.

Clipless pedals were invented by Charles Hanson in 1895 but it wasn't until the 1980s that Look (France) applied downhill ski binding technology to pedals to produce the first widely used clipless pedals. The cleat is engaged by simply pushing down and forward on the pedal, or, with some designs, by twisting the cleat in sideways. Then, instead of loosening a toestrap or pulling a lever, the cyclist releases a foot from the pedal by twisting the heel outward. The force required to release the cleat from the pedal can be altered, by a tensioning mechanism, on the pedal. Competent cyclists, or those who have been using clipless pedal systems for a while, can usually find the right amount of tensioning to balance the need for quick release of the foot in an emergency against the foot disengaging the pedal while pedaling forcefully. This is usually done by trial and error. Those new to the pedals have no guide to the amount needed to tensioning the pedal. Unlike skiing, where beginners have the bindings adjusted based on weight and ability, there is no such guide readily available for bicycle pedals. As such users risk only finding out that they have over tightened the binding mechanism when they cannot release their foot in an emergency resulting in a fall and a potential injury [2,3].

Hip fractures are common in the elderly osteoporotic population following low energy falls. They can also occur in the young adult or even the child although they often involve a high energy type injury [4]. Intra-capsular femoral neck fractures have a high tendency (10-20%) to undergo non-union or avascular necrosis of the femoral head due to its blood supply [4-6]. The main blood supply to the femoral head in the adult is through the intra-osseous and capsular vessels, emanating mainly from the medial circumflex femoral artery, a branch of the profunda femoris artery. When a displaced intra-capsular fracture occurs, the blood supply to the femoral head is compromised. In the elderly, this is dealt with by replacement of the femoral head with a metal prosthesis (hemi-arthroplasty or total hip arthroplasty), with good functional outcomes [5]. However, in the active young patient, preservation of the femoral head offers a better functional outcome although survival of the head is not guaranteed [5]. It is, therefore, accepted practice that the fracture should be reduced and fixed as quickly as possible [4,5]. Should the fracture not heal or the head not survive then a total joint arthroplasty would then be needed. This would give the patient relief of pain but return to pre-injury activity levels is not guaranteed [7].

## Conclusion

Hip fractures in the young are serious, but thankfully rare, injuries. They carry the potential for high long-term morbidity. The use of clipless pedals has become widespread over the last 20 years. Most injuries from clipless pedals are minor. We have described an extreme injury resulting from inappropriately tensioned pedals, which, to the best of our knowledge, has only been described once before in the non-English literature. It serves as a reminder of the importance of appropriate advice, especially from manufacturers and retailers, regarding the proper setup and dangers of using clipless pedals for the recreational cyclist. A system, similar to that used to adjust ski bindings, may help with the correct setup of such pedals.

## Consent

Written informed consent was obtained from the patient for publication of this case report and any accompanying images. A copy of the written consent is available for review by the Editor-in-Chief of this journal.

## Competing interests

The authors declare that they have no competing interests.

## Authors' contributions

JP was the operating surgeon and prepared a significant part of the manuscript. NP prepared a significant part of the manuscript. GD is the senior surgeon and is responsible for the ongoing management of our patient and helped in retrieving the required information for the preparation of the manuscript. Both authors have read and approved the final manuscript.
